# *MTHFR* gene C677T and A1298C polymorphisms and homocysteine levels in primary open angle and primary closed angle glaucoma

**Published:** 2009-11-09

**Authors:** Shazia Micheal, Raheel Qamar, Farah Akhtar, Muhammad Imran Khan, Wajid Ali Khan, Asifa Ahmed

**Affiliations:** 1 Department of Biosciences, COMSATS Institute of Information Technology, Islamabad-44000, Pakistan; 2 Al-Shifa Trust Eye Hospital Jhelum Road Rawalpindi-46000, Pakistan; 3 Shifa College of Medicine, Islamabad-44000, Pakistan

## Abstract

**Purpose:**

To investigate the methylenetetrahydrofolate reductase (*MTHFR)* C677T and A1298C genotypes and plasma concentrations of total homocysteine (tHcy) in Pakistani patients with primary open angle glaucoma (POAG) and primary closed angle glaucoma (PCAG).

**Methods:**

This was a prospective case-control study. A total of 295 patients (173 POAG, 122 PCAG) and 143 age- and sex-matched controls were subdivided into two ethnic groups, Punjabis (Punjab province, central Pakistan) and Pathans (North-West Frontier Province, northern Pakistan). Genotypes of the *MTHFR* C677T and A1298C polymorphisms were detected by polymerase chain reaction–restriction fragment length polymorphism (PCR-RFLP). An enzyme-linked immunosorbent assay was used to determine the total serum homocysteine (tHcy) levels. Associations were determined by logistic regression analysis.

**Results:**

Frequency distributions of genotypes and combined genotypes as well as homocysteine levels were obtained. The overall distribution of the C677T genotype was found to be significantly associated with PCAG (CC 69%, CT 21%, TT 10%; p=0.001, χ^2^=12.6), but not with POAG (CC 71%, CT 28%, TT 1%; p=0.98, χ^2^=0.02) as compared to the controls (CC 71%, CT 29%, TT 1%). The Pathan cohorts revealed no association with the disease; however, the Punjabis demonstrated a significant association with PCAG (CC 75%, CT 11%, TT 13%; p<0.001, χ^2^=17.2). PCAG in the Punjabi subjects was also significantly associated with the A1298C polymorphism (AA 43%, AC 54%, CC 3%; p<0.001, χ^2^=33.9) as compared to the controls. Combined genotype data showed no association with POAG; however, a significant association with all combined genotypes was observed in the overall PCAG subjects (p<0.05, χ^2^=20.1). This difference was particularly apparent in the TTAA and TTAC combinations that were completely absent in the control groups (p<0.05. χ^2^=49.6). Mean serum tHcy levels were found to be significantly increased in the POAG (15.2±1.28 µmol/l, p<0.001) and PCAG (20.8±4.8 µmol/l) groups as compared to the controls (10.0±0.97 µmol/l). The tHcy levels in the TT and AC genotype were significantly elevated in the PCAG group (67±12.39 µmol/l, p<0.001; 23±5.94 µmol/l, p=0.027) as compared to the controls.

**Conclusion:**

The TT and AC genotypes of *MTHFR* C677T and A1298C polymorphisms and the combined genotype TTAC were associated with PCAG in Punjabi subjects of Pakistani origin and correlated with the high serum tHcy levels seen in these patients.

## Introduction

The methylenetetrahydrofolate reductase (MTHFR) enzyme catalyzes the reduction of 5,10-methylenetetrahydrofolate to 5-methyltetrahydrofolate, the methyl donor for the conversion of homocysteine to methionine [1]. Genetic polymorphisms in the *MTHFR* gene are well established, the most extensively studied of which are C677T and A1298C single-nucleotide polymorphisms (SNPs). The C677T SNP results in a missense mutation leading to the substitution of valine for alanine at position 222 of the MTHFR enzyme, causing the synthesis of a thermolabile enzyme with a 50% reduction in activity [2-4]. The other SNP, A1298C, which is located within the COOH-terminal regulatory domain of the MTHFR, results in the substitution of glutamate for an alanine residue [5,6] and has also been associated with a mild reduction in enzymatic activity [7]. Reduced MTHFR enzyme activity is subsequently followed by increases in circulating homocysteine levels (hyperhomocysteinemia) [8].

Glaucoma is an optic neuropathy that results in progressive damage to the visual field. It is one of the leading causes of irreversible blindness in the world [9]. The two common types of glaucoma include primary open angle glaucoma (POAG) and primary closed angle glaucoma (PCAG). To date there are conflicting results regarding the association of *MTHFR* polymorphisms with glaucoma. Several studies have reported an association of the C677T polymorphism with glaucoma [10-13], whereas in other studies, particularly in European populations, this polymorphism appears to play no role in the development of glaucoma [14-17]. The relationship between the A1298C polymorphism and glaucoma has been studied in Korean [13], Swedish [14], and Japanese [15] populations, where no association has been found between the polymorphism and glaucoma.

Hyperhomocysteinemia has been observed in patients with glaucoma [17-19]. Bleich and coworkers [19] found raised plasma homocysteine levels in Caucasian glaucoma patients with the C677T polymorphism. Reports from various countries have revealed high levels of homocysteine in the aqueous humor and the serum of patients suffering from glaucoma and other ocular diseases [17,20-22]. Hyperhomocysteinemia can induce vascular injuries [23], alterations in the extracellular matrix [24], and neuronal cell death [25,26].

We recently reported a significant association of *MTHFR* C677T polymorphism with PCAG in the Pakistani population, but did not find a significant association of this mutation with POAG [27]. In the present study, we have further investigated the C677T and A1298C genotypes in POAG and PCAG in cohorts of Pakistanis, including two different ethnic groups (Pathans and Punjabis). We have also analyzed any possible association of genotypes and combined genotypes with serum homocysteine levels.

## Methods

### Patient selection criteria

Approval for the study was obtained from the Institutional Review Board/Ethics Committee. All the patients were recruited from the eye clinics of two different eye hospitals: the Christian Hospital in Taxila and Al-Shifa Trust Eye Hospital in Rawalpindi. Patients from the former hospital belonged to the Pathan ethnic group (from the North-West Frontier Province, located in the north of Pakistan), whereas from the latter the patients were Punjabi in ethnicity (from the Punjab province, located in central Pakistan). The selection criteria, the clinical tests conducted on the patients and controls, as well as the collection and processing of whole blood were the same as described previously [28]. Peripheral blood samples were obtained from patient and control groups and collected into EDTA tubes. Immediately after collection, whole blood was stored in aliquots at -20 °C until use. Informed consent was obtained from glaucoma patients and normal volunteers. Blood was obtained from a total of 295 glaucoma subjects comprising 173 patients with POAG and 122 patients with PCAG, and 143 control blood samples were also collected and processed. The POAG patients had a mean age of 50.4±1.1 years (60% males and 40% females), PCAG patients had a mean age of 52.4±1.5 years (males: 59%, females 41%) and control subjects had a mean age of 49.1±1.3 years (males: 72%, females 28%). There was no statistically significant difference between the mean age of POAG and control subjects (p=0.44) or the distribution of gender in the two groups (p=0.07). Similarly, there was no statistically significant difference in the mean age of PCAG and control subjects (p=0.09) or in the gender distribution of the two groups (p=0.053).

### Polymerase chain reaction–restriction fragment length polymorphism (PCR-RFLP)

The C677T *MTHFR* polymorphism was genotyped as previously described [27]. Briefly, we used the forward primer 5'-CCT TGA ACA GGT GGA GGC CAG-3' and the reverse primer 5'-GCG GTG AGA GTG GGG TGG AG-3'. The mixture was denatured at 95 °C for 10 min, and the PCR reaction was performed for 35 cycles under the following conditions: denaturation at 95 °C for 1 min, annealing at 65 °C for 30 s, and extension at 72 °C for 1 min and a final extension cycle of 72 °C was for 7 min. PCR products were digested with HinfI (Fermentas, Glen Burnie, MD) and analyzed on agarose gel (3%). A single fragment of 294 base pairs (bp) was identified as homozygous (CC); three fragments of 294, 168, and 126 bp were identified as heterozygous (CT); and two fragments of 168 and 126 bp were identified as homozygous (TT) genotype. The A1298C *MTHFR* polymorphism was genotyped with the help of the polymerase chain reaction (PCR) followed by digestion of the amplified product with the MboII restriction enzyme (Fermentas). Briefly, for PCR, the forward primer 5′-CTT TGG GGA GCT GAA GGA CTA CTA-3′ and the reverse primer 5′-CAC TTT GTG ACC ATT CCG GTT TG-3′ were used to amplify a 163-base pair (bp) fragment of the *MTHFR* gene. The PCR reactions were performed in a 25 μl reaction mixture containing 1× Taq Buffer (10 mM Tris-HCl, pH 9.0, 50 mM KCl, 0.1% Triton X-100, 0.01% [w/v] gelatin, 1.5 mM MgCl_2_; Fermentas), 30 pmol of each primer, 0.2 mM of the deoxynucleoside triphosphates, 1 U of Taq DNA polymerase (Fermentas), and 100 ng of genomic DNA template. The thermal profile used for the PCR amplification was the same as reported earlier for the C677T amplification except that the annealing temperature was 62 °C for 30 s. To confirm the presence of the amplicon of correct size, the PCR products were electrophoresed on 2% agarose gels (Invitrogen Corporation, Carlsbad, CA). The amplified products were then digested with the MboII restriction enzyme as per the manufacturer’s instructions. After digestion, all the fragments were resolved on 4% agarose gels. The homozygous normal allele (AA) produced 5 fragments of 56, 31, 30, 28, and 18 bp size, the heterozygous (AC) produced 3 fragments of 84, 56, and 30 bp, whereas the homozygous mutant (CC) produced 4 fragments of 84, 31, 30, and 18 bp.

### Total plasma homocysteine determination

Fasting blood samples of both the patients and control subjects were collected in plain vacutainers (Becton Dickinson, Lahore, Pakistan); the plasma was separated with the help of centrifugation and stored at -35 °C until further analysis. Total plasma homocysteine was measured using a microplate enzyme immunoassay (DRG Diagnostics, Frauenbergstrasse, Germany).

### Statistical analysis

A logistic regression and multivariate analysis was performed using Statistical Package for the Social Sciences (SPSS) 16.0 for Windows (SPSS version 16.0, Chicago, IL). The POAG, PCAG, and control data were compared using the χ^2^ test and Fisher’s exact test. The criterion for statistical significance was p<0.05.

## Results

### *MTHFR* C677T and A1298C genotype analysis

The distribution of C677T and A1298C genotypes among the controls and the patients did not deviate from those predicted by the Hardy–Weinberg equilibrium. The distribution of the *MTHFR* C677T genotype is shown in Table 1. The collective data of both ethnic groups revealed that the CC genotype was present at almost the same frequency in the three groups, i.e. 71% in controls and POAG samples and 69% in the PCAG group. Similarly, the CT genotype was also present within a statistically non-significant range, i.e. controls=29%, POAG=28%, and PCAG=21%. However, a statistically significant difference (p<0.05, χ^2^=11.8) was observed in the distribution of the TT genotype between the controls (1%) and the PCAG group (10%), with statistical analysis of the overall comparison of the genotypes also being significantly different (p<0.05, χ^2^=12.6). There was no difference between the controls and POAG group. When the data for both the ethnic groups was analyzed separately, the genotypic distribution in the Pathan patient groups (POAG & PCAG) was not significantly different from the Pathan control samples (Table 1). However, the Punjabi PCAG samples were not only significantly different from the Punjabi control samples in the overall genotype distribution pattern (p<0.05, χ^2^=17.2), but also demonstrated a statistically significant higher frequency of the mutant TT allele (p<0.05, χ^2^=10.2) and a significantly lower distribution of the CT allele (p<0.05, χ^2^=9.5) than the controls.

**Table 1:** Comparison of genotype frequencies of methyltetrahydrofolate reductase C677T polymorphism with primary open angle glaucoma, primary closed angle glaucoma and controls.

**Collective Subjects**							
**Genotype**	**Controls (143)**	**POAG (173)**	**OR (95%CI)**	**p (χ^2^)**	**PCAG (122)**	**OR (95%CI)**	**p (χ^2^)**
CC	101 (71%)	123 (71%)	0.98 (0.60-1.59)	0.98 (0.02)	84 (69%)	1.09 (0.64-1.84)	0.001 (12.6)
CT	41 (29%)	49 (28%)	1.02 (0.62-1.66)		26 (21%)	1.02 (0.62-1.66)	
TT	1 (1%)	1 (1%)	1.21 (0.12-11.7)		12 (10%)	0.06 (0.01-0.39)	
**Subjects of Pathan Ethnicity**							
**Genotype**	**Control (70)**	**POAG (96)**	**OR (95%CI)**	**p (χ^2^)**	**PCAG (61)**	**OR (95%CI)**	**p (χ^2^)**
CC	53 (76%)	70 (73%)	1.16 (0.57-2.33)	0.43 (1.7)	38 (62%)	1.89 (0.89-3.98)	0.14 (3.9)
CT	16 (23%)	26 (27%)	0.79(0.39-1.62)		19 (31%)	0.65 (0.30-1.41)	
TT	1 (1%)	0	-		4 (7%)	0.21 (0.03-1.43)	
**Subjects of Punjabi Ethnicity**							
**Genotype**	**Control (73)**	**POAG (77)**	**OR (95%CI)**	**p (χ^2^)**	**PCAG (61)**	**OR (95%CI)**	**p (χ^2^)**
CC	48(66%)	53 (69%)	0.87 (0.44-1.71)	0.54 (1.2)	46 (75%)	0.63 (0.29-1.33)	<0.001 (17.2)
CT	25(34%)	23 (30%)	1.22 (0.62-2.42)		7 (11%)	4.02 (1.62-9.89)	
TT	0	1 (1%)	0.00 (0.00-4.070		8 (13%)	0.00 (0.00-0.36)	

When the genotypic data of the *MTHFR* A1298C polymorphism was studied in the collective dataset (Table 2), a significant difference was found between the overall distribution of the genotype frequencies of PCAG and controls (p<0.05, χ^2^=9.7), with a significantly lower frequency of the mutant CC genotype in the PCAG group as compared to the controls (p<0.05, χ^2^=3.73) as well as a higher frequency of the AA normal genotype in the PCAG group when compared to the controls (p≤0.05, χ^2^=3.7). However, when the dataset was split up along the lines of ethnicity, the Pathans did not demonstrate any significant distribution in the PCAG and POAG groups when compared to the relevant controls. Contrarily, the Punjabis showed a significant difference, not only in the overall distribution of the genotypes (p<0.05, χ^2^=33.9), but also in the distribution of the different genotypes: the wild type AA allele being present at a higher frequency in the PCAG group (43%) as compared to the controls (4%), and the heterozygous AC and mutant CC alleles being present at lower frequencies (54% and 3%, respectively) as compared to the controls (73% and 23%, respectively).

**Table 2:** Comparison of genotype frequencies of methyltetrahydrofolate reductase A1298C polymorphism with primary open angle glaucoma, primary closed angle glaucoma and controls.

**Collective Subjects**							
**Genotype**	**Controls (143)**	**POAG(173)**	**OR (95%CI)**	**p (χ^2^)**	**PCAG (122)**	**OR (95%CI)**	**p (χ^2^)**
AA	20 (14%)	35 (20%)	0.64 (0.35-1.16)	0.25 (2.7)	34 (28%)	0.42 (0.23-0.78)	0.007 (9.7)
AC	97 (68%)	114 (66%)	1.09 (0.68-1.74)		76 (62%)	1.28 (0.77-2.12)	
CC	26 (18%)	24 (14%)	1.38 (0.76-2.51)		12 (10%)	2.04 (0.99-4.18)	
**Subjects of Pathan Ethnicity**							
**Genotype**	**Control (70)**	**POAG (96)**	**OR (95%CI)**	**p (χ^2^)**	**PCAG (61)**	**OR (95%CI)**	**p (χ^2^)**
AA	17 (24%)	29 (30%)	0.74 (0.37-1.48)	0.19 (3.3)	8 (13%)	2.12 (0.86-5.23)	0.26 (2.7)
AC	44 (63%)	62 (65%)	0.93 (0.49-1.75)		43 (71%)	0.71 (0.34-1.47)	
CC	9 (13%)	5 (5%)	2.68 (0.89-8.01)		10 (16%)	0.75 (0.29-1.95)	
**Subjects of Punjabi Ethnicity**							
**Genotype**	**Control (73)**	**POAG (77)**	**OR (95%CI)**	**p (χ^2^)**	**PCAG (61)**	**OR (95%CI)**	**p (χ^2^)**
AA	3 (4%)	6 (8%)	0.51 (0.13-1.94)	0.60 (1.01)	26 (43%)	0.06 (0.02-0.19)	<0.001(33.9)
AC	53 (73%)	52 (68%)	1.28 (0.63-2.55)		33 (54%)	2.25 (1.10-4.59)	
CC	17 (23%)	19 (25%)	0.93 (0.44-1.95)		2 (3%)	8.95 (2.18-36.21)	

### *MTHFR* C677T:A1298C combined genotype analysis

Combined genotypes of the C677T and A1298C polymorphisms of the *MTHFR* gene were also obtained for the collective data of both populations, as well as for the Pathans and Punjabis separately (Table 3 and Table 4). In the case of POAG, no statistically significant difference was observed when the collective data (p=0.83, χ^2^=2.8) were analyzed or when the ethnic groups were considered separately (Pathan: p=0.31, χ^2^=7.1, Punjabi: p=0.71, χ^2^=3.7; Table 3). However, when the combined genotype data of the PCAG samples were analyzed, a significant difference was observed for the collective samples (p<0.05, χ^2^=20.1), particularly for the samples containing the TT alleles (p<0.05, χ^2^=6.9; Table 4), and this was true for the TTAA as well as the TTAC genotypes (p<0.05, χ^2^=5.9, p<0.05, χ^2^=5.7, respectively). 

**Table 3:** Combined genotype analysis of methyltetrahydrofolate reductase C677T polymorphism with primary open angle glaucoma and controls.

**Combined Subjects**						
**C677T**	**A1298C**	**Controls (143)**	**POAG (173)**	**OR (95%CI)**	**p (χ^2^)**	**p (χ^2^)**
CC	AA	14 (10%)	25 (14%)	0.64 (0.32-1.28)	0.32 (2.2)	0.83 (2.8)
CC	AC	66 (46%)	79 (46%)	1.02 (0.65-1.59)		
CC	CC	21 (15%)	19 (11%)	1.39 (0.72-2.69)		
CT	AA	6 (4%)	10 (6%)	0.71 (0.26-1.94)	0.76 (0.54)	
CT	AC	30 (21%)	34 (20%)	1.08 (0.63-1.87)		
CT	CC	5 (3%)	5 (3%)	1.22 (0.37-4.01)		
TT	AA	0 (0%)	0 (0%)	--------	0.51 (1.3)	
TT	AC	1 (1%)	1 (1%)	1.21 (0.12-11.70)		
TT	CC	0 (0%)	0 (0%)	-------		
**Subjects of Pathan Ethnicity**						
**C677T**	**A1298C**	**Controls (70)**	**POAG (96)**	**OR (95%CI)**	**p (χ^2^)**	**p (χ^2^)**
CC	AA	11 (16%)	21 (22%)	0.67 (0.30-1.47)	0.09 (4.6)	0.31 (7.1)
CC	AC	33 (47%)	45 (47%)	1.01 (0.55-1.87)		
CC	CC	9 (13%)	4 (4%)	3.39 (1.05-10.85)		
CT	AA	6 (9%)	8 (8%)	1.03 (0.36-2.99)	0.68 (0.76)	
CT	AC	10 (14%)	17 (18%)	0.77 (0.34-1.79)		
CT	CC	0	1 (1%)	0.00 (0.00-5.29)		
TT	AA	0	0	--------	N/A	
TT	AC	1(1%)	0	--------		
TT	CC	0	0	--------		
**Subjects of Punjabi Ethnicity**						
**C677T**	**A1298C**	**Controls (73)**	**POAG (77)**	**OR (95%CI)**	**p (χ^2^)**	**p (χ^2^)**
CC	AA	3 (4%)	4 (5%)	0.78 (0.19-3.25)	0.88 (0.24)	0.71 (3.7)
CC	AC	33 (45%)	34 (44%)	1.04 (0.55-1.98)		
CC	CC	12 (17%)	15 (20%)	0.81 (0.36-1.85)		
CT	AA	0	2 (3%)	0.00 (0.00-2.02)	0.32 (2.2)	
CT	AC	20 (27%)	17 (22%)	1.33 (0.64-2.79)		
CT	CC	5 (7%)	4 (5%)	1.34 (0.37-4.82)		
TT	AA	0	0		N/A	
TT	AC	0	1 (1%)			
TT	CC	0	0			

**Table 4:** Combine genoype analysis of methyltetrahydrofolate reductase C677T polymorphism with primary closed angle glaucoma and controls.

**Combined Subjects**						
**C677T**	**A1298C**	**Controls (143)**	**PCAG (122)**	**OR (95%CI)**	**p (χ^2^)**	**p (χ^2^)**
CC	AA	14 (10%)	22 (18%)	0.49 (0.24-1.00)	0.05 (5.8)	0.005 (20.1)
CC	AC	66 (46%)	52 (43%)	1.15 (0.71-1.87)		
CC	CC	21 (15%)	10 (8%)	1.93 (0.88-4.20)		
CT	AA	6 (4%)	7 (6%)	1.72 (0.25-2.10)	0.43 (1.7)	
CT	AC	30 (21%)	17 (14%)	1.64 (0.86-3.12)		
CT	CC	5 (3%)	2 (2%)	2.17 (0.48-9.87)		
TT	AA	0 (0%)	5 (4%)	0.00 (0.00-0.63)	0.03 (6.9)	
TT	AC	1 (1%)	7 (6%)	0.12 (0.02-0.74)		
TT	CC	0* (0%)	0 (0%)	--------		
**Subjects of Pathan Ethnicity**						
**C677T**	**A1298C**	**Controls (70)**	**PCAG (61)**	**OR (95%CI)**	**p (χ^2^)**	**p (χ^2^)**
CC	AA	11 (16%)	5 (8%)	2.09 (0.71-6.12)	0.62 (0.97)	0.19 (9.8)
CC	AC	33 (47%)	25 (41%)	1.28 (0.64-2.56)		
CC	CC	9 (13%)	8 (13%)	0.98 (0.36-2.64)		
CT	AA	6 (9%)	2 (3%)	2.77 (0.61-12.41)	0.09 (4.8)	
CT	AC	10 (14%)	15 (25%)	0.51 (0.21-1.23)		
CT	CC	0	2 (3%)	0.00 (0.00-1.67)		
TT	AA	0	1 (2%)	0.00 (0.00-3.58)	N/A	
TT	AC	1 (1%)	3 (5%)	0.28 (0.04-2.03)		
TT	CC	0	0	---------		
**Subjects of Punjabi Ethnicity**						
**C677T**	**A1298C**	**Controls (73)**	**PCAG (61)**	**OR (95%CI)**	**p (χ^2^)**	**p (χ^2^)**
CC	AA	3 (4%)	17 (28%)	0.11 (0.03-0.38)	0.001 (17.5)	<0.001 (49.6)
CC	AC	33 (45%)	27 (44%)	1.04 (0.53-2.05)		
CC	CC	12 (17%)	2 (3%)	5.80 (1.38-24.03)		
CT	AA	0	5 (8%)	0.00 (0.00-0.61)	<0.001(21.4)	
CT	AC	20 (27%)	2 (3%)	11.13 (2.74-44.64)		
CT	CC	5 (7%)	0	----------		
TT	AA	0	4 (7%)	0.00(0.00-0.77)	N/A	
TT	AC	0	4 (7%)	0.00(0.00-0.77)		
TT	CC	0	0	----------		

When the combined genotypes of the ethnic groups were considered separately, in the Pathans no statistically significant difference was observed in the overall distribution of the alleles when compared with the controls, as well as in the case of any of the combined genotypes (Table 4). As opposed to this, when the overall data of the PCAG Punjabi samples were compared with the controls (Table 4), there was a significant difference in the distribution patterns of the combined genotypes (p<0.05, χ^2^=49.6), with almost all the genotypes showing a difference in the affected individuals compared with the controls. Both TTAA and TTAC, which were present at a frequency of 7% in the PCAG group, were absent in the controls (p<0.05, χ^2^=4.6). The CCAA combined genotype was also found to be present at a higher frequency in the Punjabi PCAG group (28%) as compared to the controls (4%), which was statistically significant (p<0.05, χ^2^=14.8). The same was the case for CTAA (PCAG=8%, controls=0%, p<0.05, χ^2^=6.2). But the CCCC and CTCC genotypes were present at a lower frequency in the affected individuals (3% and 0%, respectively) as compared to the controls (17% and 7%, respectively), which was also statistically significant (p<0.05, χ^2^=6.2 and p<0.05, χ^2^=4.3, respectively).

### *MTHFR* gene polymorphism and homocysteine concentrations

**Figure 1:**
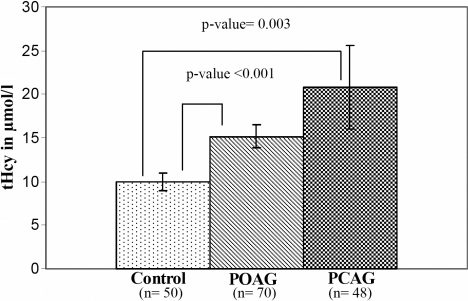
Mean plasma homocysteine levels in controls, POAG, and PCAG subjects. Error bars indicate standard error of the mean.

**Figure 2:**
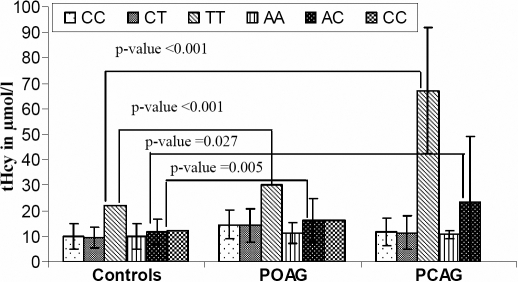
Mean plasma homocysteine levels in C677T and A1298C genotypes in controls, POAG, and PCAG samples. Error bars indicate standard error of the mean.

The associations of different genotypes with levels of tHcy were determined in a subgroup of the controls, POAG, and PCAG patients that were age- and gender matched. As shown in Figure 1, the mean value of tHcy in the POAG (15.2±1.28 µmol/l) and PCAG (20.8±4.8 µmol/l) patients was found to be significantly higher than in the controls (10.0±0.97 µmol/l). When tHcy levels were compared across different genotypes in the controls, in the case of the C677T polymorphism it was found that, compared with the normal CC genotype, the TT genotype had significantly (p<0.001) elevated tHcy levels in the controls (22 µmol/l±0.0 µmol/l) as well as in the POAG (32 µmol/l±0.0 µmol/l) and PCAG (67 µmol/l±12.39 µmol/l) groups (Figure 2). In addition, when the A1298C MTHFR genotypes were compared, both the PCAG (23±5.94 µmol/l, p<0.027) and POAG (16±1.51 µmol/l, p<0.001) groups were found to have significantly elevated levels of tHcy in the AC genotype as compared to the controls (12±1.25 µmol/l; Figure 2). When the levels of tHcy were compared against the combined genotypes, the TTAC had significantly elevated levels (67 µmol/l±12.4 µmol/l; p<0.001), whereas the rest of the combined genotypes had comparable levels of tHcy between the controls and the POAG and PCAG patients. Based upon the Wilks’ lambda, the multivariate analysis of variance analysis indicates that the tHcy levels differed significantly across the three groups (i.e. controls, POAG, and PCAG) with respect to the C677T (F=9.985, p=<0.001), but not the A1298C (F=2.543, p=0.098) genotype.

## Discussion

SNPs of the *MTHFR* gene have been shown to be associated with mild hyperhomocysteinemia. Bleich et al. [19] were the first to demonstrate an association between increased plasma homocysteine levels and POAG, which suggested evidence of its involvement in the pathogenesis of glaucomatous optic neuropathy. Subsequently, Altintas et al. [29] also demonstrated that elevation in the levels of tHcy is also associated with pseudoexfoliative glaucoma. We report here a markedly increased level of serum tHcy in Pakistani PCAG patients as compared to POAG patients and controls. To the best of our knowledge, this is the first study to show an association of hyperhomocysteinemia with PCAG. We have previously observed PCAG to be associated with the *MTHFR* C677T polymorphism in a Pakistani cohort [27]; however, at that time we did not study the A1298C polymorphism or the combined genotype frequency distributions of the two polymorphisms. Our previous study included patients and controls from a mixed population of Punjabis (from the Punjab province in central Pakistan) and Pathans (from the N.W.F.P. province in the north of Pakistan). In this study, we also investigated any correlation of the disease with the ethnic origin of the patients being studied.

Consistent with our previous report [27], we found no association of the *MTHFR* C677T genotype with POAG but a very strong association with PCAG, which is probably due to the presence of the TT genotype in the disease cohort and its complete absence from the control population (Table 1). When the samples were analyzed along ethnic lines, the Pathans did not show any of the genotypes to be associated with POAG or PCAG, but the Punjabis showed a highly significant association of PCAG with the genotypes. In the Punjabis, both the genotypes containing the mutant alleles, i.e. CT and TT, were found to be significantly associated with the disease, the former because it was present at a much lower frequency in the affected individuals and the latter because it was present at a higher frequency. The C677T C>T mutation causes an alanine>valine amino acid substitution, which results in a 55–65% loss in enzyme activity especially in individuals who are homozygous for the mutation (TT), while in the case of heterozygotes (CT) there is a 25% loss in activity compared to CC homozygous normal individuals [30,31], leading to hyperhomocysteinemia. Interestingly, when the tHcy levels were determined, a significantly higher level was observed in the PCAG subjects as compared to the POAG subjects and controls (Figure 1). This elevated tHcy level in PCAG may be mediated by the increased level of tHcy observed in the TT genotype samples of these patients (Figure 2). Although tHcy levels in the TT genotype were also higher in the controls and the POAG samples compared to the other genotypes, in the case of PCAG these levels were the highest observed, which correlates very well with the genotypic association of TT with PCAG.

PCAG patients have characteristic biometric ocular features, including a shallow anterior chamber, increased thickness of the lens, and a short axial length. It has been suggested that extracellular matrix (ECM) remodeling maybe an important determinant of the short axial length [32]. Evidence has suggested restructuring of the sclera in acute PCAG [33]. As discussed previously [27], we propose that the high level of homocysteine found in our PCAG subjects is contributing to the remodeling of the ECM of the anterior segment in these patients. Hyperhomocysteinemia has been shown to be involved in the structural remodeling of connective tissues [34]. Matrix metalloproteases (MMPs) are a class of proteases that are involved in tissue remodeling under certain pathological and physiological conditions [35]. Lui et al. [36] have reported the expression of MMP-9, which cleaves type IV collagen and is important for the remodeling of the ECM, to be decreased in the Tenon’s capsule of PCAG patients. Moreover, Seo et al. [37] have shown differential expression of MMP-2 in PCAG as compared to POAG. Recent literature supports the hypothesis that these differential expressions may be altered by variations in homocysteine levels. Kundu et al. [38] have reported MMP-2, MMP-9, and MMP-14 to be affected by homocysteine levels in the cochlea, and suggest that homocysteine induces matrix imbalance, structural changes, and oxidative stress via the extracellular signal-regulated kinase (ERK) signaling pathway [39]. Therefore, it appears logical that the enhanced activity of MTHFR results in hyperhomocysteinemia and in turn may induce differential expression of MMPs leading to tissue remodeling, thereby contributing to the disease pathogenesis of these PCAG patients.

When the A1298C mutation was studied in the different samples, no association was observed with POAG, as the frequency distribution in the combined subjects or the Pathan and Punjabi patients was not significantly different from the controls. This is in agreement with previous studies, which also did not observe any association of this polymorphism with POAG [13,15]. However, when the PCAG samples were compared with the controls, significant differences were found in the Punjabis but not in the Pathans. The Punjabi subjects had a significantly higher normal AA genotype and lower homozygous CC frequency in PCAG as compared to the controls. These results are surprising and indicate a protective role for *MTHFR* A1298C mutant alleles in PCAG patients. Although PCAG is primarily an anatomical defect, recent evidence suggests that oxidative stress may play a role in the pathogenesis of PCAG [40]. Decreased activity of the MTHFR leads not only to increased levels of plasma homocysteine, as reported here, but simultaneously to increased DNA synthesis. In this case, there is a possibility that there is an increased DNA synthesis in PCAG patients with the *MTHFR* A1298C polymorphism, that may produce a counter effect on oxidative-stress-induced DNA damage.

Our combined genotype data also does not reveal any association of the different combinations with POAG, either in the combined subjects or in the Punjabi and Pathan ethnic groups. We also did not find a single double homozygous mutant (TT/CC) in any of the controls or disease samples. This is in accordance with previous reports in which it has been suggested that both mutations cannot be simultaneously associated with glaucoma because both polymorphisms always occur in the *trans* form and not in the *cis* configuration [31]. A very strong association of the combined genotypes, particularly those containing the TT alleles of the C677T locus, was found with PCAG. When we separated the combined genotypes along the line of ethnicity, here again, only the Punjabis showed a very strong association of TT/AA and TT/AC with PCAG. A limitation of the study was that we were not able to compare the TT/AA combined genotype as there were no controls and POAG samples in this group, and so it is not possible to state whether the high levels of tHcy in the PCAG was a result of the TT genotype alone or due to the combined effect of TT/AC genotypes. It has been suggested that the A1298C polymorphism alone does not significantly affect tHcy, but does so with the combination of the 677T variant [41]. It is worth pointing out that in the Punjabis there was an unexpectedly higher frequency of the CCAA combination in the PCAG group as compared to the controls (Table 4), and even when compared with the Punjabi POAG samples (Table 3). This is because of the higher than usual frequency of the AA allele in the Punjabi PCAG samples as compared to either the controls or the Pathan samples (Table 2), which could be a result of the sample size or some other factor yet to be identified, but is definitely not a result of higher tHcy levels in this genotype.

This study is the first to report an association of the TT genotype, TT/AC combined genotypes, and high tHcy levels with PCAG. Although correlations of high homocysteine levels with the TT genotype and the TT/AC combination of MTHFR have also been reported in other diseases [31,41,42], we found the levels of tHcy to be much higher in PCAG. We suggest that this individual or combined genotype may not be sufficient to account for such high levels of tHcy in the PCAG subjects, as tHcy levels have been known to be dependent not only on *MTHFR* mutations but also on other factors, such as folate and vitamin B_12_ status [22,43]. Moreover, the nicotinamide N-methyltransferase gene has been reported to be the major genetic determinant of plasma homocysteine levels [43]. The high tHcy levels observed in this study may, however, be used as an early diagnostic biomarker for PCAG following required prospective studies. The present investigation also provides evidence of the importance of conducting such studies in different ethnic groups, as even in closely living populations such as the Pathans and Punjabis, there is clearly a difference in frequencies of genotypes and combined genotypes leading to different associations with disease.
